# Surgical Management of Congenital Nasal Pyriform Aperture Stenosis: A Case Report

**DOI:** 10.7759/cureus.21761

**Published:** 2022-01-31

**Authors:** Maho Iemura-Kashiwagi, Masahiro Kikuchi, Hideaki Okuyama, Shinzo Tanaka

**Affiliations:** 1 Otolaryngology, Head and Neck Surgery, Uji-Tokushukai Medical Center, Uji, JPN; 2 Otolaryngology, Head and Neck Surgery, Graduate School of Medicine, Kyoto University, Kyoto, JPN; 3 School of Communication Sciences and Disorders, Faculty of Medicine and Health Sciences, McGill University, Montreal, CAN

**Keywords:** stent, sublabial approach, surgical dilation, respiratory distress, nasal obstruction, congenital nasal pyriform aperture stenosis

## Abstract

Congenital nasal pyriform aperture stenosis (CNPAS) is a rare cause of respiratory distress in newborns. This paper reports a case of severe CNPAS that required endotracheal intubation immediately after birth, and eventually, surgical intervention. At birth, the width of the pyriform aperture was only 4 mm, and the patient was completely unable to breathe through his nose. We performed tracheostomy at 23 days of age and waited for the patient to grow, but at 56 days of age, the width of the pyriform aperture was not sufficient (6 mm) for the patient to breathe through his nose. Therefore, surgical dilation of the pyriform aperture by a sublabial approach was performed on day 79 after birth, and the width was increased to 14 mm. Postoperative stent placement was performed for two weeks. After the removal of the stents, the patient could finally breathe through his nose, and the postoperative course was uneventful, with no restenosis after four months. CNPAS is a rare cause of nasal obstruction, but it can cause respiratory distress in infants because they are dependent on nasal breathing. Conservative treatments are initially recommended for CNPAS; however, in severe cases where conservative treatments are ineffective, surgical treatment is recommended.

## Introduction

Congenital nasal pyriform aperture stenosis (CNPAS) is a rare cause of respiratory distress in newborns. It arises due to bony overgrowth of the nasal process of the maxilla. The pyriform aperture is the narrowest part of the nasal airway; therefore, even a minimal reduction in its diameter can cause significant problems [1]. It is often associated with solitary median maxillary central incisor, holoprosencephaly, and pituitary dysfunction; however, stenosis may also be present alone [2]. Infants are dependent on nasal breathing particularly during feeding (1); therefore, delay in initiating appropriate treatment for CNPAS puts the child at risk of respiratory distress and apnea, which, in severe cases, may lead to ischemic brain injury and death.

In cases where conservative treatments are ineffective, surgical treatment is strongly recommended due to its effectiveness [3]. However, there are few reports on the details of surgical methods and postoperative stent placement. We herein report a case of severe CNPAS who underwent surgery, had a good postoperative course, and was able to breathe through the nose as a result.

## Case presentation

Born at term from a regular pregnancy, a male newborn was admitted to our Neonatal Intensive Care Unit (NICU) immediately after birth due to respiratory distress, desaturation, and cyanosis occurring during feeding and indicated by crying. Continuous positive airway pressure (CPAP) did not improve his desaturation, and he was intubated at one-day-old. He did not have any other dysmorphic features or pituitary dysfunction. A No. 6 French suction tube (outer diameter {O.D.} 2.0 mm) (Terumo, Tokyo, Japan) could not be negotiated through either nostril, and the narrowness of the nasal cavity was notable. At this point, it was still unclear whether the narrow nasal airway was the primary cause of his dyspnea. Extubation was attempted several times, but the patient developed cyanosis, and tracheostomy was performed on day 23 after birth. Before the tracheostomy, the patient was fed through an orogastric tube. After the tracheostomy, the upper airway was closely examined using a fiberscope with a diameter of 2.2 mm and a computed tomography (CT) scan. A 2.2 mm fiberscope could barely pass the common nasal meatus and revealed that there were no choanal atresia, subglottic stenosis, vocal cord paralysis, or laryngomalacia. 

**Figure 1:**
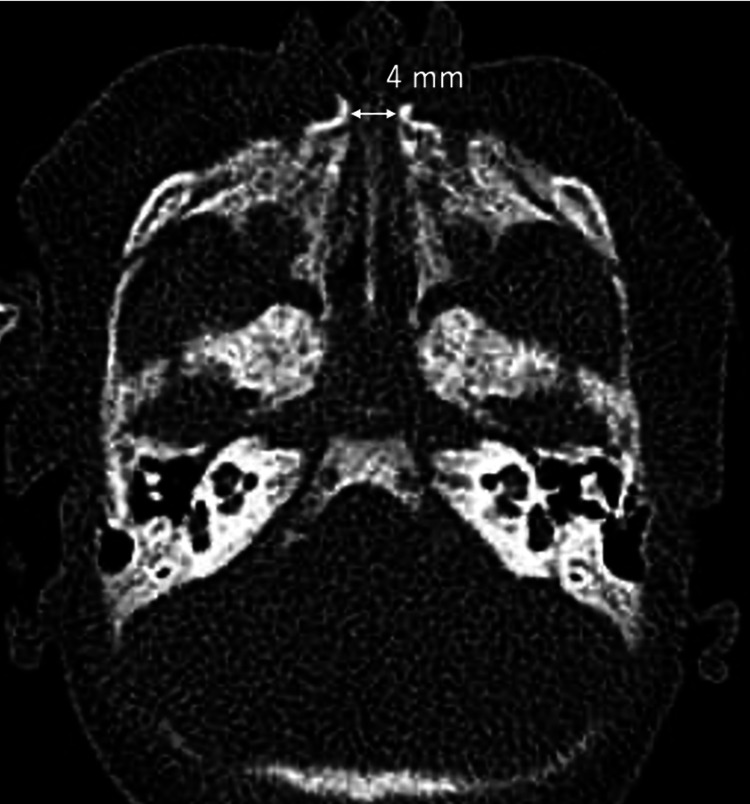
CT results on day 7 after birth. Axial CT scan on day 7 after birth showed a narrow nasal pyriform aperture. The width of the pyriform aperture was 4 mm.

The CT scan revealed a narrow nasal pyriform aperture. The width of the pyriform aperture was 4 mm (Figure 1); hence, a diagnosis of CNPAS was made as to the primary cause of his dyspnea. Conservative treatment with nasal drops and a nebulizer using saline solution was performed. We also attempted nasal stenting after intubation, but the smallest endotracheal tube with an outer diameter of 3 mm could not be inserted into the nose. The conservative treatment was continued as he grew, and the width of the pyriform aperture increased to 6 mm by day 56 after birth. However, decannulation on day 58 after birth was unsuccessful. Hence, we decided to surgically dilate the stenosis of the nasal aperture. In this period after tracheostomy, the patient also received swallowing and physical therapies. A No. 4 French nasogastric tube (O.D. 1.3 mm) (JMS, Hiroshima, Japan) was placed through the nostril on day 35, and the patient was fed through a bottle and nasogastric tube. Swallowing therapy consisted of feeding assessment and training and oral organ development assessments, including intraoral stimulation, non-nutritive sucking using a pacifier, and attempts to choose the best bottle nipple. His feeding ability was almost normal and direct breastfeeding was started on day 49. Physical therapy consisted of neurological assessment and support for motor development.

**Figure 2:**
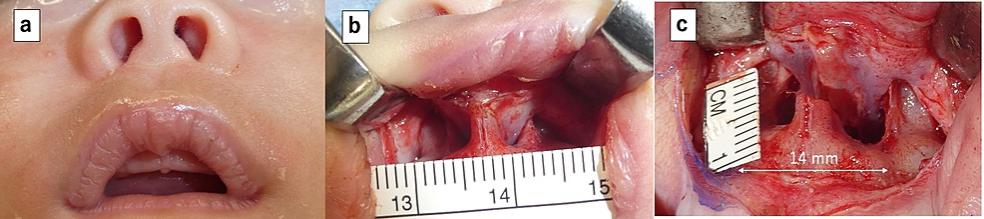
Operative findings. (a) Appearance of the nostrils before surgery. (b) A sublabial approach was performed, and the pyriform aperture and nasal process of the maxilla were exposed. (c) The nasal process of the maxilla was drilled on both sides with diamond burrs to widen the pyriform aperture from 6 to 14 mm.

The patient underwent surgery under general anesthesia on day 79 after birth. Using a sublabial approach with a gingivobuccal sulcus incision, the pyriform aperture and nasal processes of the maxilla were exposed (Figure 2). After the nasal mucosa of the nasal floor and inferior nasal meatus were elevated and preserved, the nasal process of the maxilla was drilled on both sides using 2.0 and 3.0 mm diamond burrs to widen the pyriform aperture from 6 to 14 mm (Figure 2c).

**Figure 3:**
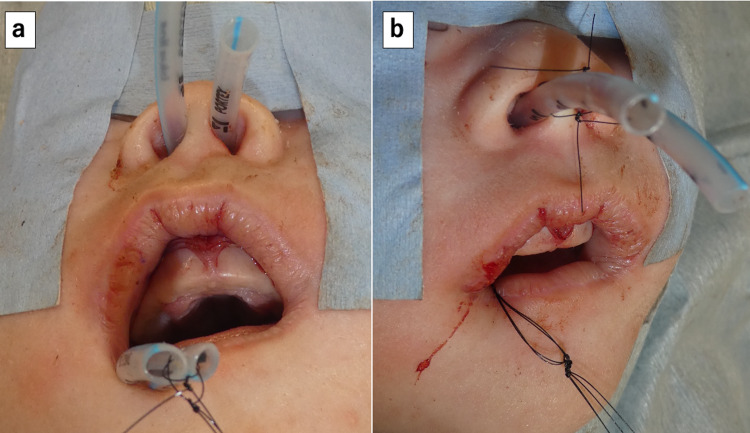
Stent placement after dilation of the nasal pyriform aperture. An endotracheal tube with an O.D. of 4.2 mm was placed in each nasal cavity as a stent. (a) The tips of the two tubes were taken out through the mouth once to be sutured to each other with nylon thread. Then, both tubes were pulled out of the nasal cavity until the sutured tips were positioned at the posterior end of the nasal septum. (b) Thereafter, both tubes were sutured in front of the anterior nostril using nylon thread.

An endotracheal tube for infants (internal diameter (I.D.) 3.0 mm, O.D. 4.2 mm) (Smiths Medical Japan, Tokyo, Japan) was placed in each nasal cavity as a stent. To keep the tubes in position, we tied each at two points, in front of and behind the nasal septum. The position was confirmed endoscopically via the oral cavity, and the tubes were finally fixed with a length of 4.5 cm in the nasal cavity (Figure 3). Antibiotics were used only postoperatively to prevent infection.

**Figure 4:**
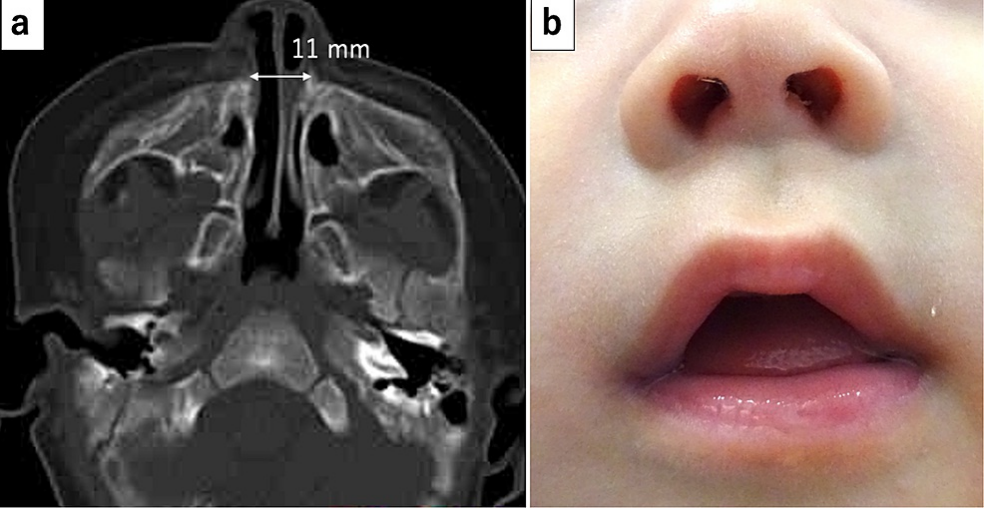
Postoperative findings. (a) Postoperative axial CT scan on the 22nd day postoperatively. The width of the pyriform aperture was 11 mm. (b) Appearance of the nostrils one month after removal of the stents.

On the 11th postoperative day, the tracheostomy tube was removed, and the tracheostomy closed spontaneously within a few days. Both stents were successfully removed on the 14th postoperative day. An ulcer was formed at the columa nasi due to the pressure of the nylon thread, but it completely healed approximately one month after the removal of the stents (Figure 4). The postoperative CT imaging 22 days after surgery showed that the pyriform aperture was 11 mm in width, as shown in the axial image at the level of the inferior nasal meatus in Figure 4a. After the stent removal, the patient was provided with direct breast as well as bottle feeding. His milk intake gradually increased, and he was discharged 32 days after surgery (111 days after birth). At a four-month follow-up, the baby had no symptoms.

## Discussion

The pyriform aperture is the narrowest part of the nasal airway; therefore, even a minimal reduction in its diameter can cause significant problems [1]. The differential diagnosis for nasal obstruction in newborns includes posterior choanal atresia, nasopharyngeal encephalocoele, nasal tumors, and CNPAS [4]. Posterior choanal atresia is the most common cause of congenital nasal obstruction in newborns, with an incidence of one in 8000. Among differential diseases for upper airway obstruction in newborns, CNPAS is not mentioned [5,6], and hence, it is considered as a rare condition. 

CNPAS is a rare cause of nasal obstruction but can be completely cured with treatment and should not be overlooked. Delay in initiating appropriate treatment puts the child at risk of respiratory distress and apnea, which, in severe cases, may lead to ischemic brain injury and death [7]. The presence of respiratory distress, including noisy breathing, desaturation, and cyclical cyanosis, together with feeding difficulties and failure of the suction tube to pass through the nostrils are potential indicators of pyriform aperture stenosis [4]. The diagnosis of pyriform aperture stenosis can be made via CT scan, by obtaining thin (1.5-3.0 mm) contiguous axial sections in a plane parallel to the anterior hard palate. The normal range of the width of the pyriform sinus in the age group of 0-6 months is 8.8-17.2 mm (median width = 13.5 mm) [8]. CNPAS is diagnosed when the pyriform aperture width is less than 11 mm in a term infant [9]; however, it has recently been suggested that symptomatic cases may be narrower [2,9]. There have been few reports of such CNPAS cases that require intubation management or tracheostomy after birth [10-14]. Initial treatment in symptomatic patients is aimed at establishing an adequate airway and conservative treatments, including the use of nasal suctioning, nasal decongestants, and room air humidification [4]. If these initial conservative treatments alone cannot resolve the signs of respiratory distress or impaired feeding, the next recommended treatment is nasal stenting [10]. An endotracheal tube, such as a Portex tube (I.D. 2.5-3.5 mm, O.D. 3.5-4.8 mm) is used as a stent and is left for one to several months [10,15], relieving the symptoms until the newborn grows and helping with dilation of the nasal airway. However, the tube sometimes causes nose bleeding and pressure necrosis of the septal mucosa and cartilage; therefore, Abbeele et al. argued that nasal stenting should be avoided [16]. Instead, they recommend a conservative treatment involving nasal steroids and topical decongestants for up to two weeks, and surgery if symptoms remained.

The patient in the present study was a severe CNPAS case with a pyriform aperture width of only 4 mm at birth. We also attempted nasal stenting after intubation, but the smallest endotracheal tube with an O.D. of 3 mm could not be inserted into the nose. Initially, we had waited for the development of the patient’s nostrils and acquisition of mouth breathing, but the pyriform aperture width was 6 mm on day 56 and the patient was not able to breathe unassisted on day 58. Although nasal stenting was not tested, the possibility of insertion of the smallest tube (O.D. 3 mm) was low. Hence, the patient’s CNPAS was still severe on day 58. Since there was no report of patients with CNPAS who were under intubation management or tracheostomy and were cured without nasal stenting or surgery, we performed the surgery on day 79. Retrospectively, we could have performed the surgery much earlier after the tracheostomy.

Surgical enlargement of the pyriform aperture is generally reserved for those who fail conservative management or nasal stenting, particularly patients experiencing ongoing dyspnea, cyanotic episodes, and failure to thrive [4]. Drilling of the nasal process of the maxilla by a sublabial approach is less invasive than the transnasal approach and is most common in newborns [17]. In the current case, we took care to elevate the nasal mucosa of the nasal floor and the inferior nasal meatus so as not to puncture the mucosa before drilling. In addition, care was taken not to damage the nasolacrimal duct during bone drilling. We believe that it is important to leave the nasal mucosa intact and perform sufficient bone drilling to prevent restenosis.

After surgery, stent placement is recommended to prevent restenosis and granulation. In addition to endotracheal tubes, soft silastic nasal stents and standard nasal conformers, usually employed in cleft surgery, are used for stents [3,18]. Botti et al. reported that stent removal at four days after the first surgery caused restenosis in the nasal cavities and relapse of respiratory distress and the patient eventually required a second surgery [18]. Although the duration of stenting is variable [17], it is recommended that stents be left in place for approximately one to two weeks [16,18,19]. In the current case, the stent was placed for two weeks, and no restenosis was observed four months postoperatively. Mild ulceration of the nasal vestibule developed due to pressure from the nylon thread used for stent fixation, but the ulcer improved spontaneously; therefore, two weeks seemed to be an appropriate period for stent placement.

CNPAS is often associated with other anomalies such as solitary median maxillary central incisor, pituitary dysfunction, and holoprosencephaly [2]. The presence of comorbidities, such as craniofacial dysmorphisms, neurologic anomalies, and airway anomalies, has also been reported to reduce the success rate of surgery [20]. The patient in the present study had no congenital complications other than CNPAS. The stents were removed after two weeks, and the postoperative course was uneventful, with no restenosis in four months.

## Conclusions

This paper reported a case of severe CNPAS requiring surgical intervention to dilate the pyriform aperture and postoperative stent placement. Two weeks after the surgery, the stent was successfully removed, and the patient could breathe through his nose. Posterior choanal atresia is the most common cause of congenital nasal obstruction in newborns, but it is important to recognize that there is another rare congenital condition called CNPAS. In cases where conservative treatments are ineffective, surgical treatment is strongly recommended because infants rely on the nasal airway.
